# Genome-wide identification of the jumonji C domain- containing histone demethylase gene family in wheat and their expression analysis under drought stress

**DOI:** 10.3389/fpls.2022.987257

**Published:** 2022-08-25

**Authors:** Xinhua Wang, Cuili Pan, Jiaohui Long, Shuangyu Bai, Mingming Yao, Jiajing Chen, Gang Sun, Yalei Fan, Zhangjun Wang, Fenglou Liu, Caixia Liu, Qingfeng Li

**Affiliations:** ^1^School of Agriculture, Ningxia University, Yinchuan, China; ^2^State Key Laboratory of Crop Gene Exploration and Utilization in Southwest China, Sichuan Agricultural University, Chengdu, China

**Keywords:** wheat, JmjC gene family, drought stress, expression analysis, genome-wide identification

## Abstract

Methylation and demethylation of histone play a crucial role in regulating chromatin formation and gene expression. The jumonji C (JmjC) domain-containing proteins are demethylases that are involved in regulating epigenetic modification in plants. In our study, the JmjC genes in *Triticum aestivum* L., *Triticum turgidum* L., *Triticum dicoccoides* L., *Triticum urartu* L., and *Aegilops tauschii* L. were identified. Phylogenetic relationship and colinearity analysis revealed that the wheat JmjC genes were conserved in A, B, and D subgenomes during evolution. *Cis*-acting elements analysis showed that elements related to stress response, hormone response, and light response were found in wheat JmjC genes. The expression of JmjC genes was affected by tissue types and developmental stages, and members of the same subfamily tended to have similar expression patterns in wheat. They also showed a unique expression pattern in root during PEG (Polyethylene glycol) treatment. In conclusion, comprehensive analysis indicated that three members (*Tr-1A-JMJ2*, *Tr-1B-JMJ2*, and *Tr-1D-JMJ2*) might be regulated by several hormones and function in the early stages of drought stress, while eight members (*Tr-1B-JMJ3*, *Tr-4B-JMJ1*, *Tr-7A-JMJ1*, etc.) displayed a significantly high expression after 24 h of PEG treatment, indicating a role in the later stages of drought stress. This research presents the first genome-wide study of the JmjC family in wheat, and lays the foundation for promoting the study of their functional characterization in wheat drought resistance.

## Introduction

Histone methylation and demethylation regulate plant development and resistance to biotic and abiotic stresses (Klose and Zhang, 2007). In eukaryotes, nucleosome, the basic structural unit of chromatin, is formed by 146 bp of DNA wrapped around an octamer nucleus composed of histones H2A, H2B, H3, and H4 (Holliday, 1987; Klose et al., 2006; Tsukada et al., 2006). Modification of histones, including methylation, acetylation, phosphorylation, and ubiquitination, could affect the conformation of chromatin, thus regulating DNA replication, transcription, and repair (Kouzarides, 2007; Huang et al., 2019). Histone methylation mainly occurs at K4, K9, K27, K36, K79, R2, R8, R17, R26 of H3 and K20, R3 of H4 (Greer and Shi, 2012). Lysine residues can be monomethylated, dimethylated, or trimethylated, while arginine can be monomethylated, symmetrically dimethylated, or asymmetrically dimethylated (Berr et al., 2011; Zheng and Chen, 2011). Histone demethylation is a reversible process regulated by demethylase (He et al., 2021). There are two classes of histone lysine demethylases in eukaryotes. Lysine specific demethylase 1 (LSD1) is a flavin adenine dinucleotide cofactor-specific reversal of monomethylation or dimethylation and can only remove monomethylation and dimethylation of H3K4, but not trimethylation of trimethyl lysine (Shi et al., 2004). The jumonji C domain-containing demethylases (JMJ-C) use divalent ferrous ions (Fe [II]) and α-ketoglutarate (α-KG) as cofactors to remove monomethylation, dimethylation, and trimethylation from multiple sites of histone lysines (Klose et al., 2006).

As one of the most important staple food crops in the world, wheat (*Triticum aestivum* L., AABBDD, 2n = 42) is a significant source of starch and energy. The production of wheat is significantly affected by biotic and abiotic stresses. The JmjC domain-containing proteins, as histone demethylases, play a crucial role in response to stress and light by modifying a series of associated transcription factors or phenotype-specific genes. For instance, in *Arabidopsis*, At4g20400 mediated H3K4 demethylation of a key promoter (FLOWERING LOCUS T, FT) and an FT homolog (TWIN SISTER OF FT, TSF) of flowers and inhibited their expression, thereby inhibiting flower transformation (Yang et al., 2010); JMJ12 was involved in the regulatory response of the light signal transduction pathway by regulating H3K27me2 and H3K27me3 demethylation (Lu et al., 2011); JMJ30 and JMJ32 removed H3K27me3 of the SnRK2.8 and activated its expression to mediate Abscisic acid (ABA) response during root development (Wu et al., 2019); AtJMJ16 regulated the level of H3K4me3 in senescence-associated genes (SAGs) and caused leaf senescence (Liu et al., 2019); Histone H3K4 demethylase AtJMJ17 and histone H3K9 demethylase JMJ27 played crucial roles in response to drought stress (Huang et al., 2019; Wang Q. et al., 2021). In rice, Os03g0151300, as a H3K4me3 demethylase, inhibited flower transition (flowering time) under long daylight conditions (Yokoo et al., 2014); H3K4me3 demethylation of OsJMJ703 participated in the resistance to drought stress in rice (Song et al., 2018). Most genes of the *BrKDM5* subfamily were dramatically up-regulated under drought stress (He et al., 2021). In barley, HvPKDM7-1 as a H3K4 demethylase homolog, was significantly increased under drought stress (Papaefthimiou and Tsaftaris, 2011). So far, the JmjC domain-containing histone demethylase gene family has been identified and studied at the genome-wide level in *Arabidopsis*, rice (Lu et al., 2008), maize (Qian et al., 2019), soybean (Han et al., 2016), *Gossypium raimondii*, *Gossypium arboreum*, *Gossypium hirsutum*, and *Gossypium barbadense* (Sun et al., 2021), respectively. However, the knowledge of their distribution, evolution, and function is still limited in wheat.

Polyploidization is widely believed to be an important driving force of evolution and diversification (Juery et al., 2021). Wheat is a heterozygous hexaploid species with AA, BB, and DD genomes. It was widely believed that wheat is formed through two polyploidization events. The first polyploidization event is the hybridization of diploid *Triticum monococcum* L. (AA) and *Aegilops speltoides* (BB) to form heterotetraploid *Triticum dicoccoides* (AABB) in about 0.5 million years ago. The second is the cross of *Triticum dicoccoides* (AABB) with *Aegilops tauschii* (DD) to create heterozygous hexaploid wheat (AABBDD) in about 10,000 years ago (Huang et al., 2002; El Baidouri et al., 2017; Li et al., 2022). Gene duplication at the genome level occurred during the polyploidy process in wheat. Identification and analysis of JmjC family genes in wheat and its wild relatives is of great significance for studying their evolution and function.

In this study, we performed a comprehensive analysis of JmjC genes, including basic features, conserved structural domains, chromosome distribution, phylogenetic relationship, *cis*-acting elements of the promoters, and expression profiles to reveal their distribution, evolution, and function in wheat. The response of wheat JmjC genes to drought stress at the seedling stage was studied using the real-time quantitative polymerase chain reaction (qPCR). The result laid a foundation for the exploration and utilization of JmjC genes for wheat drought resistance breeding.

## Materials and methods

### Genome-wide identification of jumonji C genes

The whole genome data of wheat^1^ was used to obtain the possible sequences of the JmjC gene family members. To identify all the possible JmjC genes in wheat, both Hidden Markov Model (HMM) search and Basic Local Alignment Search Tool (BLAST) were performed. The reviewed JmjC protein sequences of *Arabidopsis* were obtained from the UniProt database^2^ and used as seeds to query potential JmjC candidates *via* BLASTP with a threshold of *e*-value = 10**^––^**^5^. The HMM of JmjC (PF02373) was downloaded from Pfam.^3^ HMMER 3.3.1^4^ was used to construct HMM profiles in wheat for detection of JmjC genes with the default setting. Candidate sequences obtained from the two methods were manually checked to confirm the homology. Meanwhile, the redundant sequences of JmjC gene family members were removed. The NCBI-CDD databases^5^ were used to verify the conserved structural domains, removing incomplete sequences from the conserved structural domains. The basic properties of JmjC proteins were analyzed using the online software ExPASy.^6^ Subcellular localization was performed using the online tool PSORT Prediction.^7^

### Phylogenetic analysis

Multiple sequence comparisons of *Triticum aestivum* L., *Oryza sativa* L., and *Arabidopsis* JmjC proteins were performed using the online tool ClustalW.^8^ Then a phylogenetic tree was constructed using the maximum likelihood method (ML) of MEGA 7.0 with bootstrap parameters set 1000. The phylogenetic tree was constructed using FigTreev1.4.4 software^9^ for embellishment.

### Chromosomal distribution and collinearity analysis

Gene annotation files of wheat and gene IDs of the JmjC gene family were used to visualize chromosomal positions by TBtools (Chen et al., 2020). For collinearity analysis in wheat, comparisons between pairs of the three genomes were performed by all-against-all BLASTP searches (e-value = 10^–5^) using their proteome sequences, respectively. For Interspecies collinearity analysis, comparisons were performed using the proteome sequences of A, B, and D genome as queries against those of wheat ancestral relatives. The collinearity pairs of JmjC genes were labeled in red. MCScanX was used to detect tandem repeat genes and homologous relationships of genes in wheat. The evolution of the JmjC gene family in wheat and its relatives were analyzed by covariance analysis and collinearity analysis, and the results were visualized by the TBtools.

### Structural features analysis

Analysis and visualization of exon/intron structures of wheat JmjC genes using TBtools. The conserved structural motif of proteins in the JmjC gene family were predicted using MEME online software^10^ with a repeat setting of One Occurrence Per Sequence, a width of 6-50 amino acids, and a maximum motif of 10. The secondary structure prediction and analysis of JmjC proteins were performed using SOPMA.^11^ The tertiary structure of JmjC protein was predicted using SWISS-MODEL,^12^ an online software based on the homology modeling method.

### *Cis*-acting elements analysis

The genomic sequence of the upstream 2 kb of the JmjC genes in the whole wheat genetic data was intercepted using TBtools, and the *cis*-acting elements were predicted and manually simplified using the PlantCARE promoter prediction database.^13^ The *cis*-acting elements were visualized and mapped using the online website GSDS2.0.^14^ The heat map of *cis*-acting elements in the JmjC gene family was drowned using Origin2017 software.

### Gene expression analysis by RNA-seq

RNA-Seq data of five tissues (root, stem, leaf, spike, and seed) of Chinese spring wheat were retrieved from the Wheat Exp database,^15^ and the transcript abundance values of wheat JmjC genes were assessed using Tpm (transcripts per million reads) values. The wheat JmjC gene expression heat map was plotted using Origin2017 software.

### RNA extraction and quantitative RT-PCR

In this experiment, the common wheat Chinese spring was subjected to 20% PEG6000 drought stress using hydroponics until the material was at the third developed leaf stage. Samples were taken at 0, 1, 3, 6, 12, and 24 h after stress and stored frozen in liquid nitrogen to –80°C immediately after sampling. Total RNA was extracted from samples under PEG stress by TransZol reagent and reverse transcribed into cDNA for qPCR analysis according to the Novozymes Reverse Transcription Kit (R223). The Tr-actin gene (AY423548) was used as the internal reference gene, and the NCBI Primer-Blast^16^ was used based on the wheat JmjC gene members and the CDS sequence of the internal reference primer to design the qPCR primers (Supplementary Table 1). Reaction system formulation, 2 × ChamQ SYBR qPCR Master Mix 10 μl, PCR Forward Primer (10 μM) 0.4 μl, PCR Reverse Primer (10 μM) 0.4 μl, cDNA 4 μl ddH_2_O 5.2 μl, total 20 μl. PCR reaction conditions, 95°C 90 s, 95°C 5 s, 60°C 15 s, 72°C 20 s, 40 Cycles (Tm: 65°–95°C). The qPCR was performed using Novozymes RT-PCR reagents, and three biological replicates were set for each sample. The relative expression of target genes was calculated according to the 2^–△△^*^Ct^* method (Livak and Schmittgen, 2001), the calculations were analyzed, and graphs were produced using Excel software.

## Results

### Identification of the jumonji C gene family in wheat

A total of 24 JmjC genes were identified and analyzed comprehensively. The primary characteristics of wheat JmjC genes were analyzed, including isoelectric point (pI), molecular weight (Mw), instability coefficient (II), aliphatic amino acid index, hydrophilic index, and subcellular localization prediction. The amino acid length of Tr-JMJ proteins ranged from 806 (*Tr-6A-JMJ1*) to 1,904 (*Tr-7B-JMJ1*). Furthermore, the molecular weight ranged from 91,352.06 to 213,277.91 Da, denoting that the molecular weights and isoelectric points of the wheat JmjC gene family members differed significantly (Supplementary Table 2). In addition, subcellular predictions showed that 18 wheat JmjC gene family members were located in the nucleus, which was consistent with the occurrence of histone demethylation.

### Identification of two wheat jumonji C gene subfamilies in phylogenetics

**FIGURE 1:**
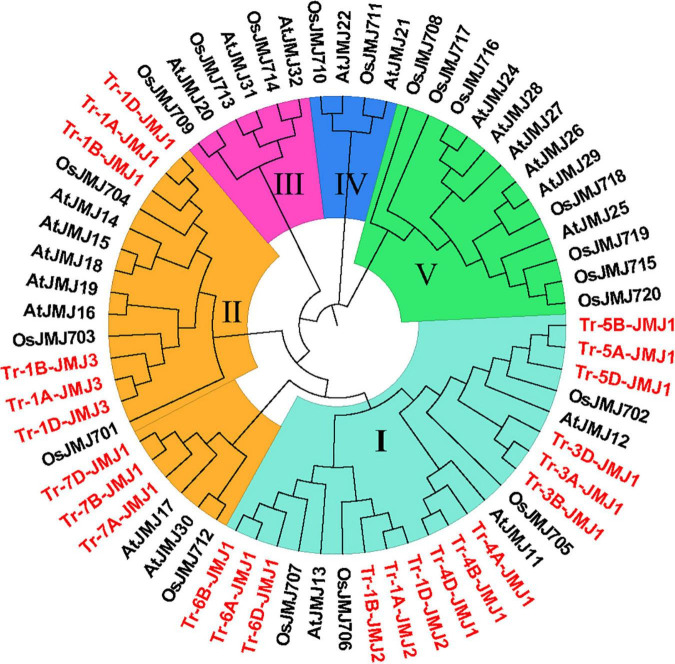
The phylogenetic tree of JmjC proteins of wheat, rice, and *Arabidopsis.* The genes in red are wheat JmjC genes. I–V represent the five subfamily according to evolutionary relationships and domain composition (I, KDM4/JHDM3; II, KDM5/JARID1; III, JmjC domain-only; IV, JMJD6; V, KDM3/JHDM2). Tr represents *Triticum aestivum* L., Os represents *Oryza sativa* L., and At represents *Arabidopsis_thaliana*.

To search the phylogenetic relationships of wheat JmjC family genes, a rootless phylogenetic tree was constructed by multiple sequence alignment of full-length protein sequences and JmjC structural domain sequences in wheat, rice, and *Arabidopsis*. Based on the results, the histone demethylases containing the JmjC domain were classified into five subfamilies: KDM3/JHDM2, KDM4/JHDM3, KDM5/JARID, JMJD6, and JmjC domain-only. The 24 JmjC genes of wheat were only distributed in two subfamilies. KDM4/JHDM3 subfamily was named subfamily I and contained 15 members. KDM5/JARID subfamily was named subfamily II and contained nine members (Figure1).

### The distribution and evolution of jumonji C family genes

**FIGURE 2:**
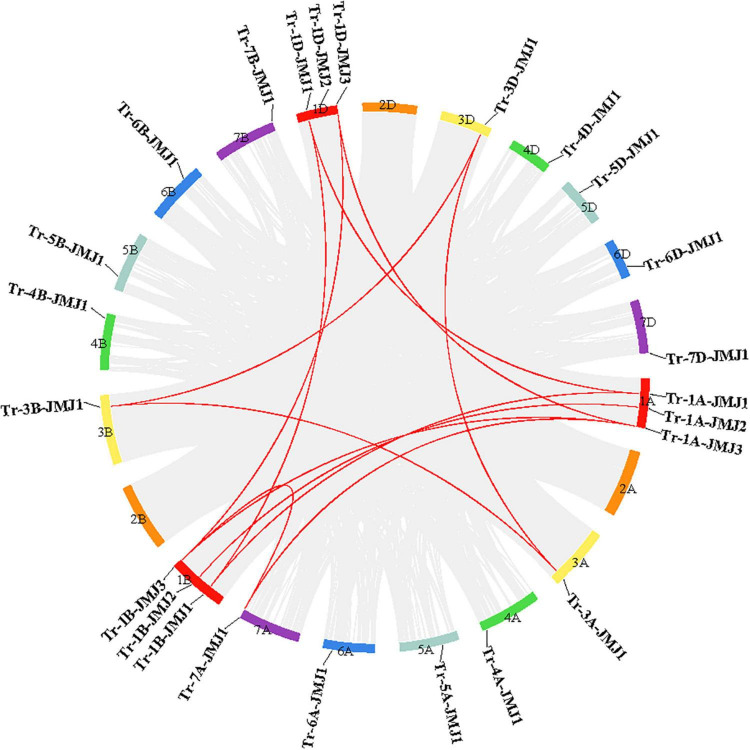
Collinearity analysis of JmjC gene in wheat. The colored boxes represent seven homologous group chromosomes of wheat. Collinear genes pairs are linked by gray lines whereas collinear JmjC genes are shown in red.

Based on the information of wheat genome annotation, we analyzed the chromosomal distribution of the 24 JmjC genes. Results showed that JmjC genes were randomly distributed on 18 of the 21 wheat chromosomes. As shown in Figure 2, chromosomes 1A, 1B, and 1D each contain three JmjC genes, chromosomes 3A-7A, 3B-7B, and 3D-7D each possess only one JmjC gene. The interchromosomal collinearity analysis in wheat revealed that there was no tandem duplication of JmjC family genes. However, twelve collinear JmjC genes pairs were found, including three pairs in chromosome 1A\1B, two pairs in both chromosomes 1A\1D and 1B\1D, and one pair in chromosomes 1A\7A, 3A\3B, 3A\3D, 1B\7A, and 3B\3D (Figure 2).

**FIGURE 3:**
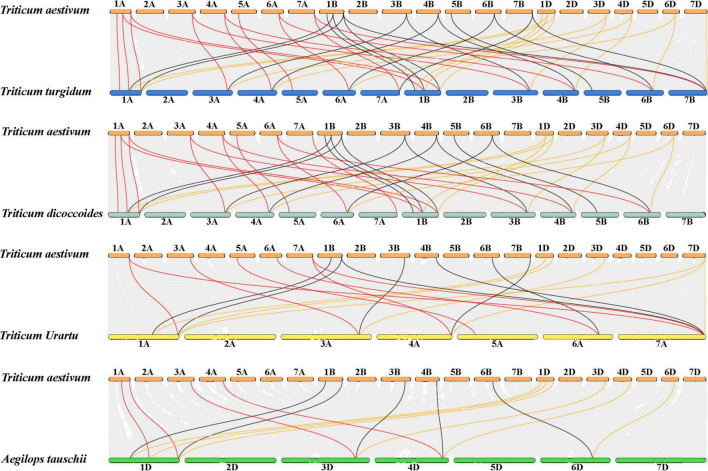
Collinear analysis of *Triticum aestivum* (AABBDD), *Triticum turgidum* L. (AABB), *Triticum dicoccoides* (AABB), *Triticum urartu* (AA), and *Aegilops tauschii*. (DD). Collinear genes pairs are linked by gray lines whereas collinear JmjC genes between A, B and D genome in wheat and its ancestral relatives are shown in red, black and yellow lines, respectively.

To further explore the evolution of the JmjC family genes, collinearity analysis was performed between wheat and its relatives: *Triticum turgidum* L., *Triticum dicoccoides*, *Triticum urartu*, and *Aegilops tauschii*. Results revealed that 16 JmjC genes in *Triticum turgidum* L. were identified as homologous to 23 JmjC genes in wheat, 15 JmjC genes in *Triticum dicoccoides* to 20 JmjC genes in wheat, 7 JmjC genes in *Triticum urartu* to 15 JmjC genes in wheat, 6 JmjC genes in *Aegilops tauschii* to 15 JmjC genes in wheat, respectively (Figure 3 and Supplementary Table 3). The three subgenomes (A, B, and D) displayed extensive homology, and there was a favorable collinearity of JmjC genes in wheat and their diploid or heterotetraploid ancestors, indicating their high conservation in evolution. The variation of JmjC family genes was also found during the evolution of wheat. For instance, Tr-5D-*JMJ1* was only found in chromosome 5D of wheat rather than *Aegilops tauschii* containing D-genome, and had no colinear gene pairs in *Triticum turgidum* L., *Triticum dicoccoides*, and *Triticum urartu*. It was speculated that *Tr-5D-JMJ1* was a newly formed gene during the evolution of wheat. *Tr-5B-JMJ1* of wheat chromosome 5B had no colinear gene pairs with both 5A and 5D in *Triticum turgidum* L., *Triticum dicoccoides*, *Triticum urartu*, and *Aegilops tauschii. Tr-7B-JMJ1* showed colinearity with *Tr-7A-JMJ1* and *Tr-7B-JMJ1* in *Triticum turgidum* L., but there was no colinear gene pair in *Triticum dicoccoides* (AABB). In addition, no homologs of the *Tr-1A-JMJ1* of *Triticum urartu* (AA), and *Tr-7D-JMJ1* of *Aegilops tauschii* (DD) had no colinear gene in wheat. This might be caused by translocation, duplication, and deletion of chromosomes and genes during the evolution of wheat and its relatives.

### Structural features of jumonji C family members in wheat

**FIGURE 4:**
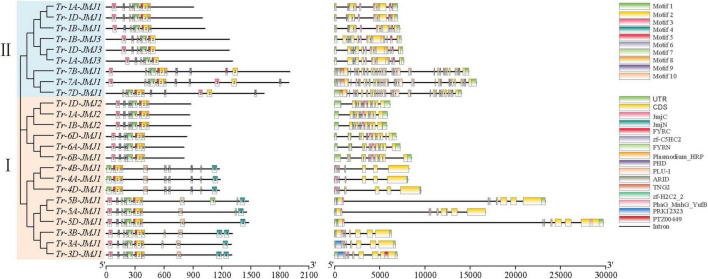
Conservative motif (left) and genes structure (right) of JmjC in wheat. The phylogenetic tree (left) was constructed by the maximum likelihood method (ML). Amino acid sequences (middle), where the 10 conserved motifs are indicated as different colored rectangles. Gene structure map (right), where CDS, UTR, and diversed domains are indicated as different colored rectangles.

The number and structures of introns and exons of genes play a crucial role in biological evolution. To understand the structural diversity, the exon-intron structures of JmjC family genes were analyzed. The number of exons of wheat JmjC genes ranged from 7 to 33 (Figure 4). The arrangement and number of exons/introns and the length of exons were consistent among the JmjC homologous genes. Meanwhile, JmjC family members were clustered into six main subfamilies according to the evolutionary clades. It could be concluded that members of the same subfamily tended to have similar gene structure, but it differed between subfamilies.

The conserved motifs composition and their positions of the 24 JmjC family proteins were further analyzed (Figure 4). Ten conserved motifs were predicted and the amino acid sequences of their conserved motifs were listed (Supplementary Figure 1). Proteins of the same subfamily showed a similar motif composition and distribution patterns, with Tr-1A-JMJ1/Tr-1B-JMJ1/Tr-1D-JMJ1 containing the fewest motifs of 7, and Tr-7A-JMJ1/Tr-3B-JMJ1/Tr-5B-JMJ1 containing the most motifs of 12. There were seven relatively conserved motifs (motif 1, 2, 3, 5, 6, 7, and 9) the 24 JmjC family proteins all contained, indicating that they might play an essential role in keeping the conservative core functions. Amino acid sequences possess specific activities and biological functions by folding into specific spatial conformations. The prediction and analysis showed that JmjC family proteins of the same subfamily tended to have similar secondary structure, including alpha-helix, beta-turn, and random coil distribution (Supplementary Table 4 and Supplementary Figure 2), and tertiary structure (Supplementary Table 5 and Supplementary Figure 3).

### The *cis*-acting elements of jumonji C genes response to light and stress

**FIGURE 5:**
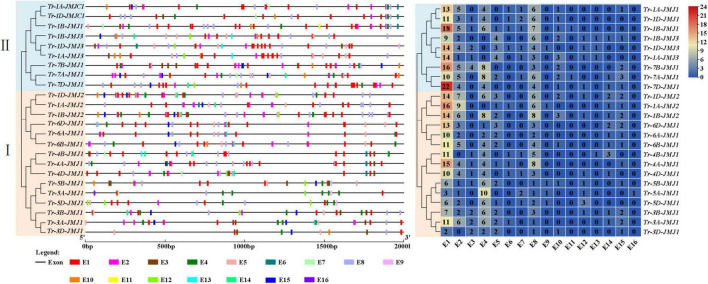
*Cis*-acting elements distribution (left) and quantity distribution (right) in wheat. E1, Light responsiveness element; E2, Abscisic acid responsiveness element; E3, Auxin-responsive element; E4, MeJA-responsiveness element; E5, Gibberellin-responsiveness element; E6, Salicylic acid responsiveness element; E7, Zein metabolism regulation element; E8, Anaerobic-induced responsiveness element; E9, Endosperm expression responsiveness element; E10, Low-temperature responsiveness element; E11, Defense and stress responsiveness element; E12, Drought responsiveness element; E13, Flavonoid responsiveness element; E14, MYB binding site element; E15, Meristematic responsiveness element; E16, DRE element.

A total of 45 *cis*-acting elements were identified by predicting the upstream 2 kb of JmjC genes in wheat, and they were divided into 16 categories according to their functions (Figure 5 and Supplementary Table 6). The number of light response elements (E1) was the most in the upstream regions of JmjC genes. *Tr-7D-JMJ1*, *Tr-1B-JMJ1*, *Tr-7B-JMJ1*, and *Tr-1A-JMJ2* all had more than 15 E1, indicating a potential role in response to light. As expected, there were multiple response elements related to stress, especially stress to drought (Figure 5). They included hormone response elements (E2, E3, E4, E5, E6, and E13), transcription factor elements (E14 and E16), and other elements related to drought stress (E8 and E12). At the same time, the clustering results showed that members of a subfamily had similar *cis*-acting element distribution (Figure 5).

### The jumonji C genes differentially expressed in tissues and development stages

**FIGURE 6:**
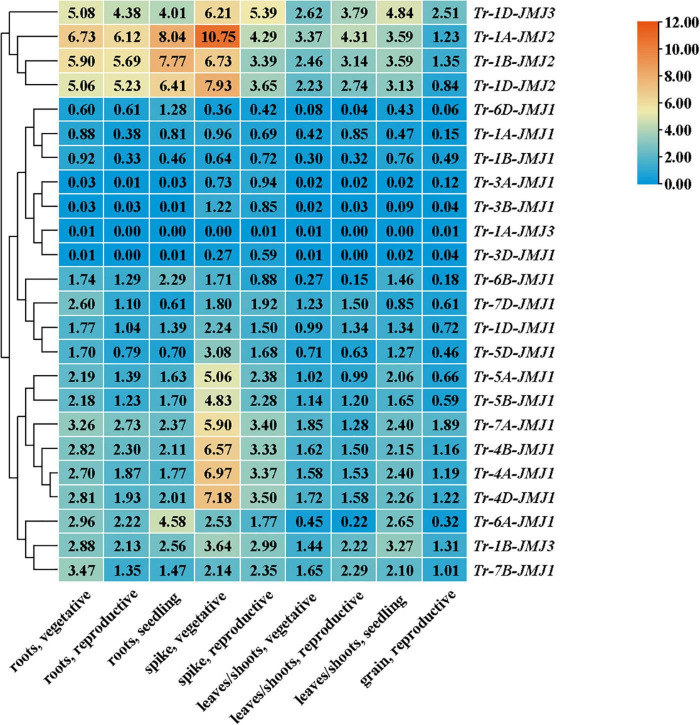
Heat map of expression profiles of wheat JmjC genes in different tissues. Phylogenetic tree (left) based on full-length protein sequences of wheat JmjC genes. Relative expression levels (middle) are indicated according to the color scale on the top right, where blue and red respectively represent low and high expression abundance. The gene names are indicated on the right. The tissue types and developmental stages are indicated at the bottom.

The expression of JmjC genes in root, stem, leaf, spike, and seed were analyzed in wheat. It revealed that the expression pattern of JmjC genes differed significantly in different tissues and developmental stages. The vegetative growth stage showed higher expression than the reproductive growth stage, and the roots and spikes displayed higher expression than leaves and grain. Importantly, the expression of *Tr-1D-JMJ2*, *Tr-1D-JMJ3*, *Tr-1A-JMJ2*, and *Tr-1B-JMJ2* was higher in all developmental periods and tissues compared with other family members (Figure 6), suggesting that they might play a more extensive and significant role in regulating the vegetative and reproductive growth of seeds, roots, leaves, and spikes. Some genes were highly expressed in the specific tissue and developmental periods, such as *Tr-4A-JMJ1*, *Tr-4B-JMJ1*, *Tr-4D-JMJ1*, etc. (Figure 6), which might play an essential role in the morphogenesis of spikes.

### The expression of jumonji C genes response to drought stress

**FIGURE 7:**
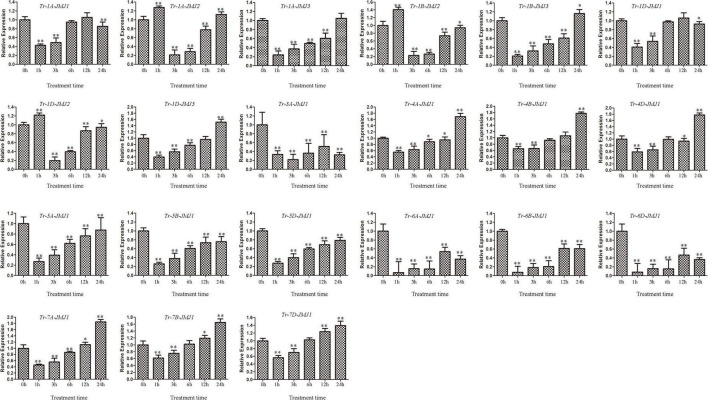
Gene expression profiles of wheat JmjC genes under polyethylene glycol (PEG) treatments. Error bars were obtained from three measurements. Symbols * and ** above the bars indicate significant differences at 0.05 and 0.01 level compared with control. These symbols above the lines represent the significance of differences between two labeled pillars.

To investigate the expression pattern of wheat JmjC genes under drought stress, the wheat Chinese Spring at the four-leaf stage was treated with PEG-3000 and the expression patterns of JmjC genes at 0, 1, 3, 6, 12, and 24 h were analyzed (Figure 7). The expression of *Tr-1A-JMJ2*, *Tr-1B-JMJ2*, and *Tr-1D-JMJ2* were significantly up-regulated at 1 h after treatment, indicating that they were responsive to drought stress. However, *Tr-4A-JMJ-C1*, *Tr-4B-JMJ-C1*, *Tr-4D-JMJ-C1*, *Tr-7A-JMJ-C1*, *Tr-7B-JMJ-C1*, and *Tr-7D-JMJ-C1* were significantly up-regulated after 24 h treatment, which might play an important role in the later stage of drought stress. In general, homologous genes tended to show similar expression patterns, pointing to their consistency in function. The results provided candidate genes for further study of drought resistance in wheat.

## Discussion

In plants, histone methylation plays a vital role in many biological processes such as transcriptional regulation and heterochromatin formation. Demethylases containing JmjC domains are essential for maintaining the balance of histone methylation levels (Kouzarides, 2007; Mirouze and Paszkowski, 2011). The JmjC gene family has been extensively studied to elucidate their functions and evolutionary history in main crops, such as rice (Sun and Zhou, 2008), maize (Qian et al., 2019), rapeseed (He et al., 2021), soybean (Han et al., 2016), and cotton (Sun et al., 2021). However, studies on the JmjC gene family in wheat are limited. In this study, we performed a comprehensive analysis of JmjC family genes in wheat, including genome-wide identification, phylogenetic relationship, gene structure, motif structure, and expression profile analysis in tissues and under drought stress. We identified 24 JmjC family genes in wheat and divided them into KDM4/JHDM3 and KDM5/JARID subfamilies according to the motif structures. Previous studies have shown that *AtJMJ17* of *Arabidopsis thaliana* and *OsJMJ703* of rice, which belonged to KDM5/JARID1 subfamily, had functions in drought resistance (Song et al., 2018; Huang et al., 2019).

Gene duplication within the genome would lead to functional differences in genes and promote the generation of new genes, which plays an important role in environmental adaptation, biological evolution, and new speciation (Conant and Wolfe, 2008). For example, the AP2/ERF and WRKY gene families are extended mainly through whole-genome multiplication, fragment duplication, and tandem duplication (Jiang et al., 2013; Li et al., 2018). In this study, a total of 12 pairs of collinearity genes were detected in wheat JmjC family genes, all of which were located between A, B, and D genomes, indicating that the JmjC genes were very conserved during the evolution of wheat, and there were genes redundancy between JmjC genes in different chromosome sets. No tandem repetition events occurred in wheat JmjC genes, which further proved that the JmjC genes were conserved in wheat evolution. What’s more, collinearity analysis of the ancestral species in Triticeae also revealed the evolution and possible origin of JmjC genes in wheat, which has certain significance for the evolution of species and gene evolution.

The JmjC domain-containing histone demethylase gene family has an important role in regulation of gene expression in plant developmental stages and responses to biotic and abiotic stresses (Han et al., 2016). RNA-seq data were used to detect the expression patterns of JmjC genes in different wheat tissues, and qPCR was used to detect the expression level of JmjC genes under drought stress. We found that wheat JmjC genes had different spatial expression patterns in different tissues and developmental periods. Still, their expression level in roots and spikes was higher than that in other tissues (Figure 7), indicating that JmjC plays an essential role in the development of roots and spikes. Expression analysis under drought stress revealed that JmjC genes were all differentially expressed compared with the control (0 h), indicating that they were sensitive to drought induction. At the beginning (1 h) of the drought stress, 18 genes tended to be down-regulated, while only 3 up-regulated. It is speculated the down-regulation of JmjC members might play a role in protecting the expression and function of genes related to more fundamental life activities. We had examined the gene expression patterns at six time points (0, 1, 3, 6, 12, and 24 h) of drought stress. However, previous studies revealed that some genes could reach peak expression after 0.5 h or less of stress, so more studies were needed to determine whether the peak still existed within 1 h. Meanwhile, JmjC members as demethylases, responded to drought by altering the methylation status of other genes, which could either activate or inhibit gene expression. The relationship between drought and methylation should be determined according to the methylation status of specific genes and loci.

Tissue and temporal expression analysis identified three groups of homologous genes that were highly expressed in roots and induced by drought stress. Combined with subcellular localization prediction, Tr-1A/1B/1D-JMJ2 were located in nucleus, which might function in the demethylation of specific sites and regulate the gene replication and transcription at an early stage (about 1 h of drought stress). Tr-7A/1B/7D-JMJ1 were also located in nucleus, but play a role at a relatively late stage (about 24 h of drought stress). Tr-4A/4B/4D-JMJ1 was located in endoplasmic reticulum, which might function in the demethylation of specific mRNA sequences and regulate their stability and translation. The homologous genes in other plant species is another important clue and basis for predicting the potential functions of JmjC genes. JmjC domain protein JMJ705, belonged to the subfamily I along with Tr-1A/1B/1D-JMJ2 and Tr-4A/4B/4D-JMJ1 (Figure 1), mediated the removal of histone H3 lysine 27 trimethylation (H3K27me3) and functioned in defense-related gene activation and low energy stress tolerance in rice (Li et al., 2013; Wang W. et al., 2021). In *Arabidopsis*, H3K27me3 demethylase genes also played key roles in response to environmental stress during plant development (Yamaguchi and Ito, 2021). Another JmjC family gene JMJ706 encoded H3K9 demethylase and mainly participated in the regulation of floral organogenesis and development (Sun and Zhou, 2008; Liu et al., 2014).

The function of JmjC genes is related to *cis*-acting elements in the upstream promoter region, which can infer the characteristics of JmjC family members. In this study, there were 45 types of *cis*-acting elements in the promoter region of wheat JmjC family genes, among which a large number of stress-related response elements such as salicylic acid and MeJA were distributed. It could speculate that JmjC family genes were involved in the drought response process in wheat. Except for DRE elements, the other drought-related response elements were distributed in the three groups of homologous genes (Tr-1A/1B/1D-JMJ2, Tr-4A/4B/4D-JMJ1 and Tr-7A/1B/7D-JMJ1). The distribution characteristics of *cis*-acting elements are related to the regulatory function of JmjC genes (Figure 5). For instance, Tr-1A-JMJ2, Tr-1B-JMJ2 and Tr-1D-JMJ2 all had Abscisic acid responsiveness element, Gibberellin-responsiveness element, Anaerobic-induced responsiveness element, and MYB binding site element. Tr-1B-JMJ2 and Tr-1D-JMJ2 contained MeJA-responsiveness element and Drought responsiveness element (MBS), whereas TR-1A-JMJ2 lacked the two elements, indicating the regulatory differences between Tr-1B-JMJ2, Tr-1D-JMJ2, and Tr-1A-JMJ2.

In conclusion, a comprehensive genome-wide analysis of the JmjC gene family was conducted in wheat. Phylogenetics and collinearity analysis revealed that the JmjC gene family is homologous in A, B, and D genome of wheat and its ancestral relatives. Expression analysis and functional prediction indicated that JmjC genes might play a significant role in wheat drought stress. Tr-1A/1B/1D-JMJ2, Tr-4A/4B/4D-JMJ1, and Tr-7A/1B/7D-JMJ1 as candidate genes provide an essential reference for further studies of the regulation of drought stress in wheat.

## Data availability statement

The datasets presented in this study can be found in online repositories. The names of the repository/repositories and accession number(s) can be found in the article/Supplementary material.

## Author contributions

XW and QL designed the research. XW participated in all bioinformatic analysis, experiments, analysis, and wrote the manuscript. JL, SB, MY, JC, GS, YF, ZW, FL, and CL conceived and directed the study and helped to draft the manuscript. XW, CP, and QL revised and improved the draft. All authors contributed to the article and approved the submitted version.

## Footnotes

1
https://wheat-urgi.versailles.inra.fr

2
https://www.uniprot.org/

3
https://pfam.xfam.org/

4
http://hmmer.org/

5
https://www.ncbi.nlm.nih.gov

6
https://web.expasy.org

7
http://psort1.hgc.jp/form.html

8
https://www.genome.jp/tools-bin/clustalw

9
http://tree.bio.ed.ac.uk

10
http://meme-suite.org/

11
https://swissmodel.expasy.org

12
https://swissmodel.expasy.org

13
http://bioinformatics.psb.ugent.be/webtools

14
http://gsds.gao-lab.org/

15
http://www.wheat-expression.com

16
https://www.ncbi.nlm.nih.gov/tools/primer-blast/
