# Supplementary material for: Genome-wide identification of the jumonji C domain- containing histone demethylase gene family in wheat and their expression analysis under drought stress
Source: Front Plant Sci. 2022 Aug 25;13:987257. doi: 10.3389/fpls.2022.987257 (PMC9453444; doi:10.3389/fpls.2022.987257)
Supplement: Supplementary file 1 [file Table_1.DOCX]

Supplementary Material

**Table S1 The RT-qPCR primers of the wheat JmjC genes**

| **Gene name** | **Gene ID** | **Direction of primers** | **5'--3'** | Tm（℃） | Product（bp） |
| --- | --- | --- | --- | --- | --- |
| *Tr-5B-JMJ1* | TraesCS5B02G110600.1 | F | CCAGAAGGAAAGGAAGGGACT | 59.1 | 195 |
|  |  | R | ATTGCTGAGTGCTTGGTGCTT | 60 |  |
| *Tr-1D-JMJ2* | TraesCS1D02G199000.1 | F | CAGTGGCTTCAAAATACGGCA | 61.3 | 82 |
|  |  | R | 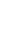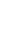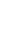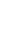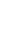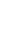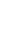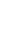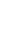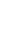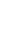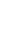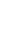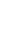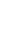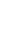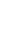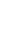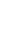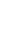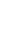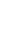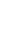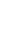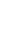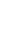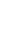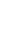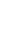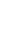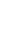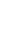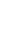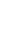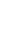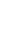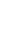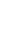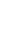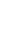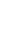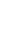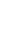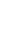TCCTTCATCAGCACGACACC | 58.8 |  |
| *Tr-1A-JMJ1* | TraesCS1A02G133800.3 | F | 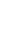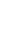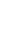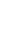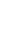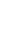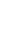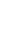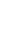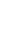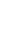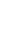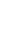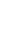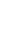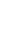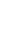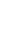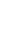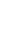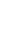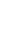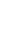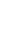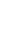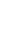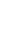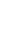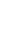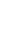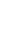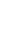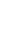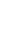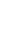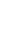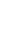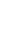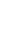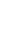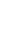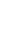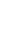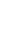GTCAACTGGGCAAACCTTCT | 56.6 | 183 |
|  |  | R | GAAACTCCTCCACAGCCACC | 59.1 |  |
| *Tr-1A-JMJ2* | TraesCS1A02G195700.1 | F | GGAGACTTGAGAGGATGCGTT | 58.3 | 265 |
|  |  | R | TTGGTGGGACAATACACAGGG | 60.3 |  |
| *Tr-4B-JMJ1* | TraesCS4B02G317400.1 | F | CGTCCCCGTATCTTCTGCTT | 59.3 | 269 |
|  |  | R | CCCATCTGTGTCGTCCAATCT | 59.5 |  |
| *Tr-3A-JMJ1* | TraesCS3A02G412700.1 | F | CAGTGTATTGGTGGCTCAGGT | 57.4 | 176 |
|  |  | R | AGTGGCTCCTTCACAGTCTC | 53.6 |  |
| *Tr-4D-JMJ1* | TraesCS4D02G314000.1 | F | CACAGAGACAACCGAGACGAA | 58.3 | 163 |
|  |  | R | CAAGCAGAAGATACGGGGAC | 57.2 |  |
| *Tr-1D-JMJ3* | TraesCS1D02G439600.1 | F | AACTTGCTGATGCTCGTCCT | 57.2 | 115 |
|  |  | R | CTACAGTGCGTGTCGTTTCG | 57.5 |  |
| *Tr-5A-JMJ1* | TraesCS5A02G109400.1 | F | TCAAACGGGAGGTGCCAGA | 61.8 | 242 |
|  |  | R | CTTGCCGAAGTGGAGGTAGT | 56.9 |  |
| *Tr-3D-JMJ1* | TraesCS3D02G407400.1 | F | TCCATTTCAGGGAAGCCAACA | 62.7 | 103 |
|  |  | R | CCCAGTTTCACAGCCCAATC | 60.1 |  |
| *Tr-1D-JMJ1* | TraesCS1D02G124700.1 | F | GGTGGCTGTGGAGGAGTTTC | 59.1 | 489 |
|  |  | R | CTGCCGCTGATTGCTTTGTT | 61.3 |  |
| *Tr-6D-JMJ1* | TraesCS6D02G248400.1 | F | TGGAGATACGCACATTCAGCA | 59.9 | 100 |
|  |  | R | GCGAGCACCCATTTTCATAAGT | 61.1 |  |
| *Tr-7A-JMJ1* | TraesCS7A02G566000.1 | F | TGGTTCTGGAGGAGAGATGGA | 59.2 | 305 |
|  |  | R | CGAGCGGAGGTGATAAACAG | 58.3 |  |
| *Tr-5D-JMJ1* | TraesCS5D02G124200.1 | F | 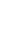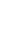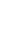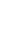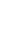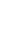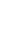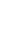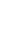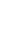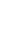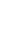GATGCTGTATGTGGCGATGCT | 60.9 | 92 |
|  |  | R | GCTTGCCGAAGTGGAGGTAG | 59.9 |  |
| *Tr-7B-JMJ1* | TraesCS7B02G492100.1 | F | TGTTTGCCTTGAGCACTGGA | 59.8 | 179 |
|  |  | R | TTTTAGATGGCGTGGGGATGT | 61.8 |  |
| *Tr-1A-JMJ3* | TraesCS1A02G430400.3 | F | ACCCTACCTCAGAATCCACCA | 58.3 | 160 |
|  |  | R | TCGCAATCTCCTCATCAAGCA | 61.5 |  |
| *Tr-1B-JMJ2* | TraesCS1B02G210400.1 | F | ATGGTGGAGGGACGGAACTG | 61.7 | 329 |
|  |  | R | TTGGTGGGACAATACACAGGG | 60.3 |  |
| *Tr-1B-JMJ3* | TraesCS1B02G465100.2 | F | ACCCTACCTCAGAATCCACCA | 58.3 | 160 |
|  |  | R | TCGCAATCTCCTCATCAAGCA | 61.5 |  |
| *Tr-7D-JMJ1* | TraesCS7D02G544800.1 | F | GTGGTCGCAAAGAACGGTG | 59.4 | 232 |
|  |  | R | GGGTGTGGCGGTAAAGTAAG | 57.4 |  |
| *Tr-1B-JMJ1* | TraesCS1B02G146900.2 | F | ATGGTTTCCTCCTCCGCCTC | 62.7 | 103 |
|  |  | R | CCGTTCTGACTCGCATTCTC | 58 |  |
| *Tr-3B-JMJ1* | TraesCS3B02G447100.1 | F | AAACGACCCTGTTGTCCCCT | 60.6 | 348 |
|  |  | R | ATTGTCCCCCTTCACCCTTC | 60.1 |  |
| *Tr-4A-JMJ1* | TraesCS4A02G416800.1 | F | GGGCATCAACACAGAGACAAC | 57.5 | 172 |
|  |  | R | CAAGCAGAAGATACGGGGAC | 57.2 |  |
| *Tr-6A-JMJ1* | TraesCS6A02G266300.3 | F | TGGAGATACGCACATTCAGCA | 59.9 | 100 |
|  |  | R | GCGAGCACCCATTTTCATAAGT | 61.1 |  |
| *Tr-6B-JMJ1* | TraesCS6B02G293600.2 | F | CAGCATTGTGTCCGTTGGTC | 59.2 | 178 |
|  |  | R | GGCACTTCCTAATCTCTTCCTCA | 59.6 |  |
| *Tr-actin* | AY423548.1 | F | GGGGCTATTCCTTCACCACA | 59.7 | 149 |
|  |  | R | CCCGTCAGGCAGTTCGTA | 57.2 |  |

**Table S2 Physicochemical properties and subcellular localization of JmjC protein in wheat**

| **Gene name** | **Gene ID** | **Lenth(aa)** | **The instability index (II)** | **Aliphatic index** | **GRAVY** | **Molecular Weight (ku)** | **Isoelectric point（PI）** | **Subcellular localization** |
| --- | --- | --- | --- | --- | --- | --- | --- | --- |
| *Tr-1A-JMJ1* | TraesCS1A02G133800.3 | 907 | 53.64 | 67.13 | -0.556 | 102217.54 | 6.24 | nucleus |
| *Tr-1A-JMJ2* | TraesCS1A02G195700.1 | 866 | 57.69 | 65.77 | -0.546 | 96944.31 | 8.26 | nucleus |
| *Tr-1A-JMJ3* | TraesCS1A02G430400.3 | 1311 | 59.08 | 68.04 | -0.560 | 146025.39 | 7.95 | nucleus |
| *Tr-1B-JMJ1* | TraesCS1B02G146900.2 | 1023 | 54.40 | 70.67 | -0.521 | 115598.94 | 6.19 | nucleus |
| *Tr-1B-JMJ2* | TraesCS1B02G210400.1 | 880 | 56.52 | 67.16 | -0.517 | 98211.78 | 8.42 | nucleus |
| *Tr-1B-JMJ3* | TraesCS1B02G465100.2 | 1277 | 59.95 | 68.19 | -0.572 | 141693.31 | 7.40 | nucleus |
| *Tr-1D-JMJ1* | TraesCS1D02G124700.1 | 998 | 54.80 | 67.17 | -0.603 | 112753.39 | 6.45 | nucleus |
| *Tr-1D-JMJ2* | TraesCS1D02G199000.1 | 876 | 58.66 | 65.35 | -0.553 | 97913.37 | 8.48 | nucleus |
| *Tr-1D-JMJ3* | TraesCS1D02G439600.1 | 1276 | 59.27 | 66.78 | -0.589 | 141790.42 | 7.96 | nucleus |
| *Tr-3A-JMJ1* | TraesCS3A02G412700.1 | 1298 | 49.31 | 68.95 | -0.554 | 142724.06 | 8.92 | nucleus |
| *Tr-3B-JMJ1* | TraesCS3B02G447100.1 | 1309 | 51.24 | 69.42 | -0.545 | 144171.85 | 8.88 | nucleus |
| *Tr-3D-JMJ1* | TraesCS3D02G407400.1 | 1303 | 51.15 | 70.78 | -0.535 | 143265.97 | 8.90 | nucleus |
| *Tr-4A-JMJ1* | TraesCS4A02G416800.1 | 1182 | 52.93 | 72.89 | -0.487 | 129705.80 | 5.20 | endoplasmic reticulum |
| *Tr-4B-JMJ1* | TraesCS4B02G317400.1 | 1175 | 53.46 | 73.77 | -0.480 | 129754.14 | 5.14 | endoplasmic reticulum |
| *Tr-4D-JMJ1* | TraesCS4D02G314000.1 | 1175 | 54.88 | 73.20 | -0.480 | 129306.41 | 5.10 | endoplasmic reticulum |
| *Tr-5A-JMJ1* | TraesCS5A02G109400.1 | 1458 | 47.00 | 66.90 | -0.687 | 163414.84 | 6.92 | nucleus |
| *Tr-5B-JMJ1* | TraesCS5B02G110600.1 | 1476 | 46.38 | 66.59 | -0.677 | 165223.82 | 7.01 | nucleus |
| *Tr-5D-JMJ1* | TraesCS5D02G124200.1 | 1478 | 46.12 | 67.37 | -0.680 | 165366.01 | 6.92 | nucleus |
| *Tr-6A-JMJ1* | TraesCS6A02G266300.3 | 806 | 53.62 | 73.43 | -0.391 | 91376.04 | 6.98 | cytoplasm |
| *Tr-6B-JMJ1* | TraesCS6B02G293600.2 | 806 | 52.71 | 73.43 | -0.381 | 91352.06 | 6.88 | microbody (peroxisome) |
| *Tr-6D-JMJ1* | TraesCS6D02G248400.1 | 835 | 51.34 | 73.56 | -0.377 | 94614.76 | 6.65 | cytoplasm |
| *Tr-7A-JMJ1* | TraesCS7A02G566000.1 | 1894 | 52.43 | 84.87 | -0.341 | 212382.73 | 5.85 | nucleus |
| *Tr-7B-JMJ1* | TraesCS7B02G492100.1 | 1904 | 52.45 | 84.83 | -0.342 | 213277.91 | 5.78 | nucleus |
| *Tr-7D-JMJ1* | TraesCS7D02G544800.1 | 1638 | 51.81 | 86.55 | -0.340 | 184578.14 | 5.67 | nucleus |

**Table S3 Colinearity between wheat and its wild relatives**

| Chromosomes of *Triticum aestivum* | JMJC Gene Name of *Triticum aestivum* | *Triticum aestivum* JMJC gene ID | Chromosomes of *Aegilops tauschii* | *Aegilops tauschii* JMJC gene ID |
| --- | --- | --- | --- | --- |
| 1A | *Tr-1A-JMJ3* | TraesCS1A02G430400.3 | 1D | AET1Gv21018500.3 |
| 1A | *Tr-1A-JMJ2* | TraesCS1A02G195700.1 | 1D | AET1Gv20507600.3 |
| 3A | *Tr-3A-JMJ1* | TraesCS3A02G412700.1 | 3D | AET3Gv20922300.2 |
| 4A | *Tr-4A-JMJ1* | TraesCS4A02G416800.1 | 4D | AET4Gv20753100.1 |
| 1B | *Tr-1B-JMJ3* | TraesCS1B02G465100.2 | 1D | AET1Gv21018500.3 |
| 1B | *Tr-1B-JMJ1* | TraesCS1B02G146900.2 | 1D | AET1Gv20321800.13 |
| 3B | *Tr-3B-JMJ1* | TraesCS3B02G447100.1 | 3D | AET3Gv20922300.2 |
| 4B | *Tr-4B-JMJ1* | TraesCS4B02G317400.1 | 4D | AET4Gv20753100.1 |
| 6B | *Tr-6B-JMJ1* | TraesCS6B02G293600.2 | 6D | AET6Gv20679500.1 |
| 1D | *Tr-1D-JMJ3* | TraesCS1D02G439600.1 | 1D | AET1Gv21018500.3 |
| 1D | *Tr-1D-JMJ2* | TraesCS1D02G199000.1 | 1D | AET1Gv20507600.3 |
| 1D | *Tr-1D-JMJ1* | TraesCS1D02G124700.1 | 1D | AET1Gv20321800.13 |
| 3D | *Tr-3D-JMJ1* | TraesCS3D02G407400.1 | 3D | AET3Gv20922300.2 |
| 4D | *Tr-4D-JMJ1* | TraesCS4D02G314000.1 | 4D | AET4Gv20753100.1 |
| 6D | *Tr-6D-JMJ1* | TraesCS6D02G248400.1 | 6D | AET6Gv20679500.1 |

**Table S4 The protein secondary structure of JmjC protein in wheat**

| Gene ID | Gene name | Alpha helix | Extended strand | Beta turn | Random coil |
| --- | --- | --- | --- | --- | --- |
| TraesCS1A02G133800.3 | *Tr-1A-JMJC1* | 0.3091 | 0.1236 | 0.0287 | 0.5386 |
| TraesCS1A02G195700.1 | *Tr-1A-JMJC2* | 0.2705 | 0.1329 | 0.0486 | 0.548 |
| TraesCS1A02G430400.3 | *Tr-1A-JMJC3* | 0.3206 | 0.1366 | 0.0389 | 0.5038 |
| TraesCS1B02G146900.2 | *Tr-1B-JMJC1* | 0.3307 | 0.1213 | 0.0352 | 0.5127 |
| TraesCS1B02G210400.1 | *Tr-1B-JMJC2* | 0.2878 | 0.1308 | 0.0444 | 0.537 |
| TraesCS1B02G465100.2 | *Tr-1B-JMJC3* | 0.3088 | 0.127 | 0.0376 | 0.5266 |
| TraesCS1D02G124700.1 | *Tr-1D-JMJC1* | 0.336 | 0.1023 | 0.0271 | 0.5346 |
| TraesCS1D02G199000.1 | *Tr-1D-JMJC2* | 0.2949 | 0.1189 | 0.048 | 0.5383 |
| TRAESCS1D02G439600.1 | *Tr-1D-JMJC3* | 0.3082 | 0.1216 | 0.04 | 0.5302 |
| TraesCS3A02G412700.1 | *Tr-3A-JMJC1* | 0.2506 | 0.1295 | 0.0555 | 0.5644 |
| TraesCS3B02G447100.1 | *Tr-3B-JMJC1* | 0.2752 | 0.1254 | 0.0573 | 0.542 |
| TraesCS3D02G407400.1 | *Tr-3D-JMJC1* | 0.2588 | 0.119 | 0.0538 | 0.5684 |
| TRAESCS4A02G416800.1 | *Tr-4A-JMJC1* | 0.3133 | 0.1702 | 0.0627 | 0.4539 |
| TraesCS4B02G317400.1 | *Tr-4B-JMJC1* | 0.3109 | 0.1593 | 0.046 | 0.4838 |
| TRAESCS4D02G314000.1 | *Tr-4D-JMJC1* | 0.3075 | 0.155 | 0.0477 | 0.4898 |
| TraesCS5A02G109400.1 | *Tr-5A-JMJC1* | 0.2382 | 0.1078 | 0.0487 | 0.6054 |
| TraesCS5B02G110600.1 | *Tr-5B-JMJC1* | 0.2529 | 0.1092 | 0.0468 | 0.5912 |
| TraesCS5B02G163000.1 | *Tr-5B-JMJC2* | 0.2799 | 0.175 | 0.0645 | 0.4807 |
| TraesCS5D02G124200.1 | *Tr-5D-JMJC1* | 0.2674 | 0.1151 | 0.0542 | 0.5633 |
| TraesCS5D02G170100.5 | *Tr-5D-JMJC2* | 0.2858 | 0.1602 | 0.0664 | 0.4876 |
| TraesCS6A02G266300.3 | *Tr-6A-JMJC1* | 0.2658 | 0.1255 | 0.0435 | 0.5652 |
| TraesCS6B02G293600.2 | *Tr-6B-JMJC1* | 0.287 | 0.1329 | 0.0447 | 0.5354 |
| TraesCS6D02G248400.1 | *Tr-6D-JMJC1* | 0.265 | 0.1379 | 0.048 | 0.5492 |
| TraesCS7A02G566000.1 | *Tr-7A-JMJC1* | 0.524 | 0.0882 | 0.0438 | 0.3439 |
| TRAESCS7B02G492100.1 | *Tr-7B-JMJC1* | 0.5223 | 0.0893 | 0.0441 | 0.3442 |
| TraesCS7D02G544800.1 | *Tr-7D-JMJC1* | 0.537 | 0.0935 | 0.0495 | 0.3201 |

**Table S5 Prediction of protein tertiary structure of JmjC protein in wheat**

| Gene ID | Gene name | Seq Identity | GMQE | QMEAN |
| --- | --- | --- | --- | --- |
| TraesCS5B02G110600.1 | *Tr-1A-JMJ1* | 0.4317 | 0.07 | -4.66 |
| TraesCS1D02G199000.1 | *Tr-1A-JMJ2* | 0.6159 | 0.40 | -2.10 |
| TraesCS1A02G133800.3 | *Tr-1A-JMJ3* | 0.5545 | 0.40 | -2.50 |
| TraesCS1A02G195700.1 | *Tr-1B-JMJ1* | 0.6122 | 0.41 | -2.10 |
| TraesCS4B02G317400.1 | *Tr-1B-JMJ2* | 0.4199 | 0.05 | -1.76 |
| TraesCS3A02G412700.1 | *Tr-1B-JMJ3* | 0.4457 | 0.10 | -3.62 |
| TRAESCS4D02G314000.1 | *Tr-1D-JMJ1* | 0.4268 | 0.05 | -3.18 |
| TRAESCS1D02G439600.1 | *Tr-1D-JMJ2* | 0.5747 | 0.24 | -1.99 |
| TraesCS5A02G109400.1 | *Tr-1D-JMJ3* | 0.4278 | 0.07 | -4.36 |
| TraesCS3D02G407400.1 | *Tr-3A-JMJ1* | 0.4425 | 0.10 | -4.96 |
| TraesCS1D02G124700.1 | *Tr-3B-JMJ1* | 0.5554 | 0.34 | -2.59 |
| TraesCS6D02G248400.1 | *Tr-3D-JMJ1* | 0.6263 | 0.43 | -2.37 |
| TraesCS7A02G566000.1 | *Tr-4A-JMJ1* | 0.3803 | 0.11 | -5.36 |
| TraesCS5D02G124200.1 | *Tr-4B-JMJ1* | 0.4305 | 0.07 | -4.29 |
| TRAESCS7B02G492100.1 | *Tr-4D-JMJ1* | 0.3669 | 0.14 | -4.78 |
| TraesCS1A02G430400.3 | *Tr-5A-JMJ1* | 0.5736 | 0.22 | -1.81 |
| TraesCS1B02G210400.1 | *Tr-5B-JMJ1* | 0.6151 | 0.39 | -2.17 |
| TraesCS1B02G465100.2 | *Tr-5D-JMJ1* | 0.5728 | 0.25 | -2.21 |
| TraesCS7D02G544800.1 | *Tr-6A-JMJ1* | 0.4098 | 0.09 | -4.36 |
| TraesCS1B02G146900.2 | *Tr-6B-JMJ1* | 0.5554 | 0.32 | -2.97 |
| TraesCS3B02G447100.1 | *Tr-6D-JMJ1* | 0.4428 | 0.10 | -4.59 |
| TRAESCS4A02G416800.1 | *Tr-7A-JMJ1* | 0.4268 | 0.05 | -3.17 |
| TraesCS6A02G266300.3 | *Tr-7B-JMJ1* | 0.6297 | 0.44 | -2.50 |
| TraesCS6B02G293600.2 | *Tr-7D-JMJ1* | 0.6297 | 0.44 | -2.42 |

**Table S6 The statistics of cis-acting elements**

| Cis-acting elements number | Cis-acting elements type | Cis-acting elements name |
| --- | --- | --- |
| E1 | Light responsiveness element | GT1-motif |
| E1 | Light responsiveness element | G-Box |
| E1 | Light responsiveness element | Sp1 |
| E1 | Light responsiveness element | GA-motif |
| E1 | Light responsiveness element | chs-CMA1a |
| E1 | Light responsiveness element | TCT-motif |
| E1 | Light responsiveness element | Box 4 |
| E1 | Light responsiveness element | GTGGC-motif |
| E1 | Light responsiveness element | LAMP-element |
| E1 | Light responsiveness element | ATCT-motif |
| E1 | Light responsiveness element | ACE |
| E1 | Light responsiveness element | L-box |
| E1 | Light responsiveness element | TCCC-motif |
| E1 | Light responsiveness element | MRE |
| E1 | Light responsiveness element | I-box |
| E1 | Light responsiveness element | AE-box |
| E1 | Light responsiveness element | chs-Unit 1 m1 |
| E1 | Light responsiveness element | chs-CMA2a |
| E1 | Light responsiveness element | ATC-motif |
| E1 | Light responsiveness element | AAAC-motif |
| E1 | Light responsiveness element | GATA-motif |
| E1 | Light responsiveness element | 4cl-CMA2b |
| E1 | Light responsiveness element | CAG-motif |
| E10 | Low-temperature responsiveness element | LTR |
| E11 | Defense and stress responsiveness element | TC-rich repeats |
| E12 | Drought responsiveness element | MBS |
| E13 | Flavonoid responsiveness element | MBSI |
| E14 | MYB binding site element | CCAAT-box |
| E15 | Meristematic responsiveness element | CAT-box |
| E16 | DRE element | DRE |
| E2 | Abscisic acid responsiveness element | ABRE |
| E3 | Auxin-responsive element | TGA-element |
| E3 | Auxin-responsive element | AuxRR-core |
| E3 | Auxin-responsive element | TGA-box |
| E4 | MeJA-responsiveness element | CGTCA-motif |
| E4 | MeJA-responsiveness element | TGACG-motif |
| E5 | Gibberellin-responsiveness element | P-box |
| E5 | Gibberellin-responsiveness element | GARE-motif |
| E5 | Gibberellin-responsiveness element | TATC-box |
| E6 | Salicylic acid responsiveness element | TCA-element |
| E7 | Zein metabolism regulation element | O2-site |
| E8 | Anaerobic-induced responsiveness element | ARE |
| E8 | Anaerobic-induced responsiveness element | GC-motif |
| E9 | Endosperm expression responsiveness element | GCN4_motif |
| E9 | Endosperm expression responsiveness element | AACA_motif |

**Figure S1 The conserved motifs of 24 JmjC protein amino acid sequences**

**Figure S2 The distribution of Alpha helix, Beta turn, and Random coil in the secondary structure domains of the JmjC genes proteins**

**Figure S3 The tertiary structures of the JmjC genes proteins**
